# Pleiotrophin and metabolic disorders: insights into its role in metabolism

**DOI:** 10.3389/fendo.2023.1225150

**Published:** 2023-07-07

**Authors:** Cristina Ballesteros-Pla, María Gracia Sánchez-Alonso, Javier Pizarro-Delgado, Agata Zuccaro, Julio Sevillano, María Pilar Ramos-Álvarez

**Affiliations:** Departamento de Química y Bioquímica, Facultad de Farmacia, Universidad San Pablo-CEU, CEU Universities, Urbanización Montepríncipe, Boadilla del Monte, Madrid, Spain

**Keywords:** pleiotrophin, receptor protein tyrosine phosphatase β/ζ (RPTP β/ζ), metabolism, metabolic disorders, peripheral organs

## Abstract

Pleiotrophin (PTN) is a cytokine which has been for long studied at the level of the central nervous system, however few studies focus on its role in the peripheral organs. The main aim of this review is to summarize the state of the art of what is known up to date about pleiotrophin and its implications in the main metabolic organs. In summary, pleiotrophin promotes the proliferation of preadipocytes, pancreatic β cells, as well as cells during the mammary gland development. Moreover, this cytokine is important for the structural integrity of the liver and the neuromuscular junction in the skeletal muscle. From a metabolic point of view, pleiotrophin plays a key role in the maintenance of glucose and lipid as well as whole-body insulin homeostasis and favors oxidative metabolism in the skeletal muscle. All in all, this review proposes pleiotrophin as a druggable target to prevent from the development of insulin-resistance-related pathologies.

## Introduction

Pleiotrophin (PTN) is an 18-kDa neurotrophic heparin-binding factor that was simultaneously described by several groups around 1990 (1–5). *Ptn* gene encodes a basic protein of 168 amino acids, that after post-transcriptional modifications, renders the active protein composed of 136 amino acids and a 32 amino acid signal peptide (3, 5, 6). In addition, PTN maintains a highly conserved sequence among species (sequence identity >90%) and shares more than 50% sequence identity with midkine, the other member of the family (7).

*Ptn* expression is highly upregulated during embryonic development and during early cell differentiation (2, 4, 5). In the adulthood, its expression is decreased in most of the tissues except for the bone and the nervous system, where the highest expression levels are maintained (1–5). In humans, circulating PTN levels are significantly associated with advancing chronological age, what confirms a retention of pleiotrophin expression in adult tissues (8). In particular, a residual expression of pleiotrophin has been found in the adult liver, brain, adipose tissue, testis, and pancreas (9–11).

Additionally, upregulated pleiotrophin expression has been associated with biological events that involve cellular proliferation and differentiation such as tissue regeneration (12), bone repair (13), inflammatory processes (14), hypoxia (15), tumor growth (16) and angiogenesis (17). In fact, *Ptn* gene is a proto-oncogene and high levels of this cytokine are associated to perineural invasion in pancreatic cancer (18) and metastasis in prostate cancer (19). Accordingly, determination of PTN levels has been proposed as a potential tool for the diagnosis and prognosis of breast cancer (20, 21). Moreover, the expression of this cytokine has shown to be enhanced in the presence of several growth factors and cytokines, like androgens (22), TNFα and epidermal growth factor (EGF) (23), platelet-derived growth factor (PDGF) and basic fibroblast growth factor (FGF) (24), and to be downregulated in a dose-dependent manner by 1α,25-Dihydroxyvitamin D(3) (25).

Additionally, several miRNAs are also regulators of PTN expression. miR-143 represses *Ptn* expression and enhances preadipocyte differentiation (26), miR-182 downregulates PTN levels and participates in the development of endometrial receptivity (27). PTN levels are negatively correlated with the levels of miR-137 a miRNA that is decreased in hypertrophic scars (28) and of those of miR‐384 a microRNA with potential tumor suppressor activity (29). Moreover, some miRNAs (miR-499 and miR-1709) regulate *Ptn* expression by affecting the DNA methylation status of the *Ptn* gene, and hence, favor the initiation and progression of some tumoral processes (30).

From a pharmacodynamic point of view, PTN is a ligand for several receptors that are differentially expressed in the different tissues. So far, PTN has been described to interact with nucleolin (19), neuropilin-1 (31), syndecans (32, 33), integrin αvβ3 (34), integrin αMβ2 (Mac-1) (35), anaplastic lymphoma kinase (ALK) (36), and receptor protein phosphatase β/ζ (RPTP β/ζ) (37, 38). PTN interacts with the glycosaminoglycans of syndecans and induces its oligomerization affecting its interactions with other membrane receptors, what has shown to activate focal adhesion kinase and ERK1/2 (39). Similarly, PTN can also bind to the extracellular domain of RPTP β/ζ, inducing its dimerization, what inhibits the intracellular tyrosine phosphatase activity and favors the increment in the phosphorylated forms of the RPTP β/ζ substrates including β-catenin, ALK, c-Src, PKC, integrin αVβ3, p190RhoGAP and ERK1/2 (40). Although, ALK has been described as a receptor for pleiotrophin (36) it is not clear if pleiotrophin directly modulates ALK signaling or if it is a target of the PTN/RPTP β/ζ signaling pathway (41). PTN treatment induces the phosphorylation of the cytoplasmic domains of β3 of αvβ3 integrin (34). Furthermore αvβ3 forms a multi receptor complex with RPTP β/ζ and nucleolin (42). Pleiotrophin interacts with nucleolin that participates in PTN nuclear translocation what have been suggested that may modify gene expression (43). Altogether, the binding of PTN to these receptors triggers the activation of intracellular signaling cascades involved in cell migration and adhesion (19, 44, 45), cancer and metastasis (19, 44, 45), inflammation (35, 46, 47) and neurite outgrowth (2).

## Pleiotrophin in metabolic organs: implications for metabolism and disease

Metabolic diseases are characterized by the disruption of normal metabolic processes which alter the capacity to process carbohydrates, proteins or lipids. Metabolic disorders include a wide range of diseases that can be either inherited or acquired during lifetime. Metabolic syndrome is characterized by the presence of insulin resistance, central obesity, hypercholesterolemia and hypertension and poses a higher risk of developing diabetes and cardiovascular disease (48).

The role of the central nervous system (CNS) in controlling peripheral metabolism has been extensively described in the literature (49). The continuous crosstalk between the brain and peripheral organs through different molecules is key in the regulation of whole-body metabolic homeostasis (50). Recently, the link between neurodegenerative diseases and metabolic disorders has gained more relevance. It has been proposed that the different molecules involved in the crosstalk between the brain and the adipose tissue, might contribute to the development of several metabolic and/or neurodegenerative disorders (49, 51). Thus, diabetic or obese patients are prone to develop neurodegenerative disorders such as Parkinson´s or Alzheimer´s Disease (52, 53). However, the mechanisms underlying these connections remain still unclear.

PTN is a potent modulator of neuroinflammation in the CNS (14) and participates in the repair, survival and differentiation of neurons (54). PTN modulates the tyrosine phosphorylation of substrates that are involved in neuroinflammation through RPTP β/ζ, which is mainly expressed in the adult CNS in both neurons and microglia (55). As PTN signaling pathway regulates the inflammatory condition related to metabolic disorders, pleiotrophin has been postulated as a promising candidate for CNS and peripheral metabolism crosstalk (55). Moreover, recent reports have evidenced that PTN is implicated in the regulation of peripheral metainflammation, metabolic homeostasis, thermogenesis, as well as insulin sensitivity in the peripheral tissues (11, 56, 57) Furthermore, *Ptn* deletion protects against neuroinflammation, mitochondrial dysfunction, and aberrant protein aggregation in a high fat diet (HFD) induced obesity model (47).

**Figure 1:**
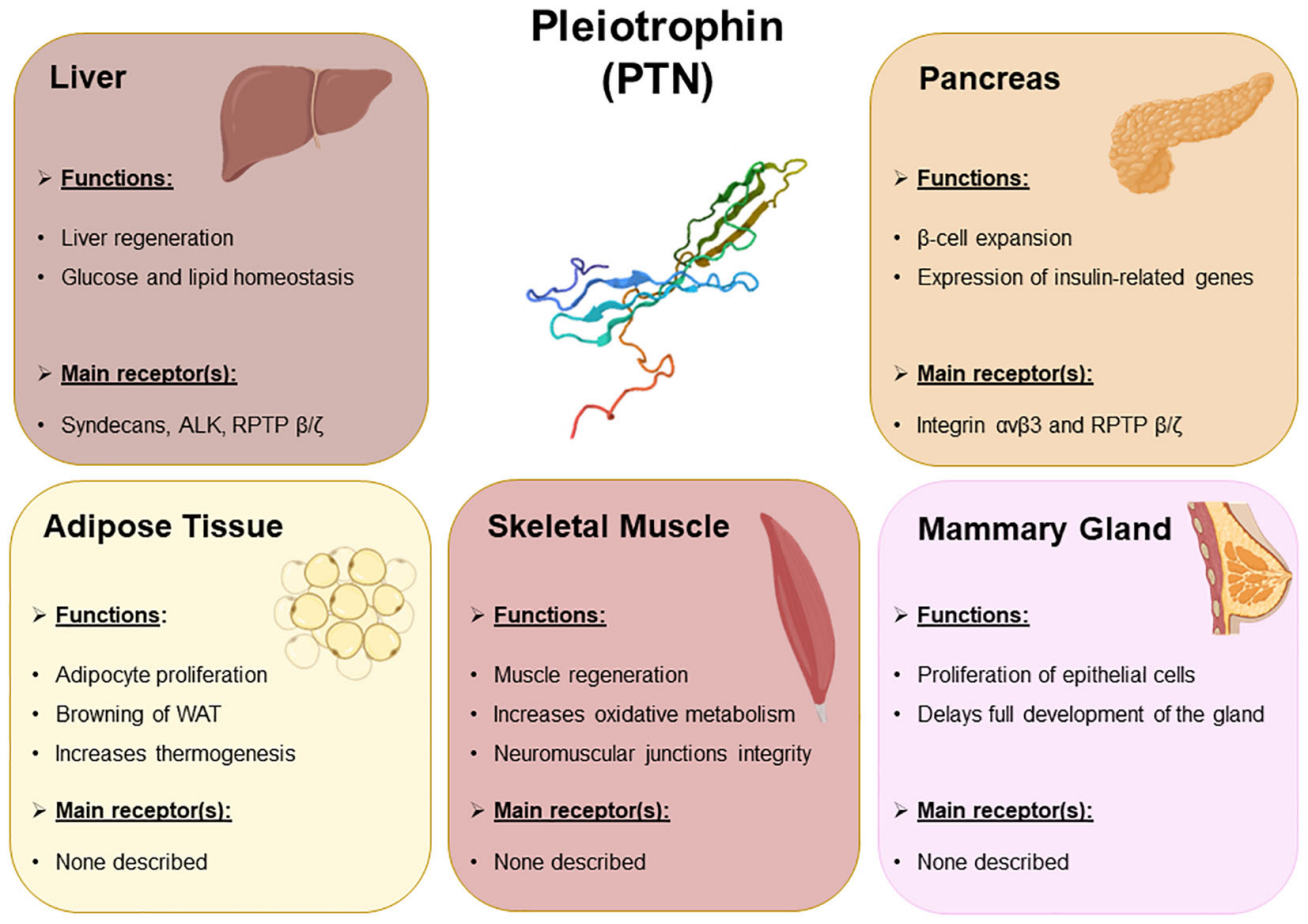
Summary of the main biological events regulated by PTN occurring in the metabolic organs. The main metabolic events regulated by PTN in liver, pancreas, adipose tissue, skeletal muscle, and mammary gland. The receptors of PTN expressed in these organs that presumably mediate PTN signaling pathways have also been included.

In this review we summarize the main roles of pleiotrophin in the development and metabolism of key metabolic organs (**Figure 1**), and how these effects may contribute to the development of metabolic disorders and more precisely, metabolic syndrome.

## Pleiotrophin in liver regeneration and metabolic regulation

The liver is the main metabolic organ of the organism and regulates energy homeostasis by modulating glucose and lipid metabolism (58, 59). Liver integrity is crucial for liver functionality. Accordingly, liver has a unique capacity to regenerate after tissue damage (60), and several cytokines and growth factors have been shown to be essential for liver regeneration (61–65). PTN exerts an antiapoptotic activity through the inhibition of caspase-3 (66) and is a potent mitogen for hepatocytes (67). In this line of evidence, PTN potentiates regeneration after partial hepatectomy (68), regulates the proliferation of bile ducts after liver injury and is overall implicated in liver regeneration and development (68, 69). In fact, after liver damage, under hypoxic conditions or in the presence of high PDGF levels (15), *Ptn* expression and secretion is induced in the hepatic stellate cells (HSCs) and once secreted, PTN exerts its mitogenic effects on hepatocytes (68, 70). Regarding the metabolic effects of pleiotrophin in hepatocytes, PTN binding to the N-syndecan activates the PI_3_K/Akt/mTORC1 pathway what increases the levels of sterol regulatory element‐binding protein 1c (SREBP‐1c) and the expression of lipogenic genes, inducing fatty acid synthesis (29).

In the liver, *Ptn* deletion in mice lowered lipid accumulation and decreased the mRNA levels of the enzymes involved in both fatty acid and triacylglyceride synthesis. Although the exposure to a HFD promoted hyperinsulinemia and insulin resistance in wild type mice, *Ptn* deletion blocks the HFD-induced hyperinsulinemia and the increment in the HOMA-IR index, and also protects against HFD-induced hepatic steatosis (57).

On the other hand, PTN has also shown to be necessary for hepatic homeostasis during pregnancy (56). Deletion of *Ptn* is associated with a defective hepatic peroxisome proliferator-activated receptor alpha (PPARα) and NUR77 activation that impairs lipid and carbohydrate metabolism. As a consequence, in the absence of pleiotrophin, a marked reduction in fatty acid, triacylglyceride and cholesterol synthesis is observed in the liver. Additionally, hepatic secretion of triacylglycerides and hepatic lipid accumulation are reduced in the pregnant *Ptn* knock out mice (56).

## Role of pleiotrophin in skeletal muscle development and function

Pleiotrophin is expressed in all types of muscle cells (smooth muscle, cardiac muscle, and skeletal muscle) and it is distributed in the basement membranes and epithelial cell surfaces (71–73). First of all, expression of pleiotrophin has been reported in intestinal smooth muscle cells (24). Secondly, *Ptn* is upregulated during *in vitro* myogenesis as well as in rat soleus muscle during regeneration after crushing. The expression levels of PTN progressively increased during the differentiation process with a peak during the fusion of myoblasts into myotubes (74). Moreover, PTN staining was localized in differentiating skeletal muscles and in the outer mesenchymal layer of the stomach and the intestine, which gives rise to the smooth muscle layer (75). PTN has neurotrophic activity and induces clustering of acetylcholine receptors (AchR) at neuromuscular junctions (NMJ). During rat embryogenesis, at embryonic day E16, PTN is located close to AchR clusters on the muscle cell surface (76). Moreover, *Ptn* is expressed in macrophages and non-myelinating glial cells (Schwann cells) which are important cell types implicated in the NMJ (77). PTN also accumulates in extracellular structures that line growing axonal processes and disappears after neurite extension has been completed (78).

During the regeneration of adult muscle, in a general inflammatory *milieu*, platelets and myogenic precursor cells start secreting various growth factors, to counteract the deleterious effects of a muscle lesion (71–73). PDGF, a chemotactic factor for satellite cells, is released by platelets from injured vessels and may contribute to the increment in the expression of PTN in the damaged area (74). Pleiotrophin not only enhances vascularization mechanisms but is equally involved in the formation of new myofibers (74). PTN mRNA and protein expression increase during the regeneration process of a crushed muscle with a peak at day 5 in the newly formed myotubes and in the activated myoblasts, being the levels restored 15 days after the damage (74).

Skeletal muscle plays a key role in metabolism, uptaking up to 80% of postprandial glucose and it is one of the key players in the development of whole-body insulin resistance (79). Moreover, muscle functions as an endocrine organ given its capacity to secrete molecules known as myokines that are released in the circulation (80). These myokines are essential for the crosstalk between the skeletal muscle and other organs, such as the adipose tissue, the brain, or the bone and may favor the development of some metabolic diseases (80). Transgenic mice over-expressing PTN in bone (*Ptn*^Tg^), under the control of the human bone specific osteocalcin promoter, exhibit higher oxidative metabolism in soleus muscle. This increment in oxidative metabolism may be the consequence of the enrichment in the number of the highly oxidative type 1 fibers, and higher mRNA levels of ATP-sensitive K^+^ channel (*Abcc8*), cytochrome c oxidase subunit IV (*Cox4i1*), and citrate synthase (*Cs*), and increased vascularization (72). Although these effects observed in these mice overexpressing PTN seem to be related to a paracrine action of this cytokine, from a metabolic point of view, a higher oxidative metabolism in the skeletal muscle may have a protective action against the development of insulin resistance and type 2 diabetes and highlights the role of this cytokine in the metabolic homeostasis in the skeletal muscle.

## Role of pleiotrophin in adipogenesis and adipose tissue browning

An impairment of adipogenesis favors the development of obesity, insulin resistance and diabetes (81). Consequently, the molecules regulating preadipocyte proliferation and adipogenesis might be targets for the treatment of these metabolic diseases. PTN is expressed in the adipose tissue (82), and differential expression of PTN was found between omental and subcutaneous adipose tissue. As increased omental adipose tissue mass has a higher risk of obesity-associated metabolic diseases, PTN expression has therefore been proposed as a possible link between obesity, diabetes, and cardiovascular diseases (82).

Pleiotrophin gene expression varies during adipocyte differentiation suggesting a possible role of PTN in adipogenesis (26, 83). Particularly, *Ptn* expression is almost undetectable in preadipocytes, increases during confluence and quickly decreases when differentiation is induced. Moreover, as *Ptn* expression is induced by ADAMTS1 (ADAM metallopeptidase with thrombospondin type 1 motif 1) via the Wnt/β-catenin pathway, the activation of ADAMTS1 could also play a role in preadipocyte differentiation (84). In fact, when brown adipocytes differentiate, the levels of *Adamts1* (84) are decreased and so the levels of *Ptn* (11), supporting the idea that PTN might be implicated in adipocyte proliferation but not in differentiation. In fact, as shown in the 3T3-L1 cell line, PTN impairs preadipocyte differentiation through the inhibition of PPARγ (Peroxisome proliferator-activated gamma) and through a crosstalk between the PTN/PI_3_K/Akt/GSK-3β/β-catenin and the Wnt/Fz/GSK-3β/β-catenin signaling pathways (83).

Some micro-RNAs that regulate adipogenesis have been involved in the development of metabolic disorders such as obesity and diabetes (85). miR-143 favors preadipocyte differentiation, as its levels begin to increase when adipocyte differentiation is induced. Furthermore, miR-143 interacts with the coding region of *Ptn* gene and it is able to repress *Ptn* expression (26). An up-regulated expression of miR-143 in association with an increased expression of *Pparγ* and *aP2* (adipocyte protein 2), two adipocyte genes involved in the pathophysiology of obesity and insulin resistance, was found in the adipose tissue of obese mice fed with HFD (86).

Recent studies using a *Ptn* knock out mice model (*Ptn*^-/-^) reveal that *Ptn* deletion is associated with decreased body weight and adiposity, altered fat distribution, impaired periovarian white adipose tissue expandability, increased catecholamine-induced lipolysis and reduced inhibitory response to insulin of lipolysis (11). Moreover, *Ptn*^-/-^ mice are glucose intolerant and develop insulin resistance in later life and preferentially oxidize fatty acids as main energy source instead of glucose both in the light and dark periods (11). In fact, *Ptn* deletion is associated with an increased conversion of T_4_ to T_3_ by deiodinase 2 (DIO2) and increased fatty acid thermogenesis in brown adipose tissue (11). Additionally, pleiotrophin deletion is associated with browning and increased expression of uncoupling protein-1 (UCP-1) and other specific markers of brown/beige adipocytes in periovarian adipose tissue of female *Ptn*^-/-^ mice (57). Therefore, it has been proposed that absence of pleiotrophin has a protective role against high fat diet-induced insulin resistance and obesity in rodents.

## Role of pleiotrophin in mammary gland development

PTN stimulates angiogenesis and promotes invasion and metastasis in breast cancer. Thus, PTN protein was purified for the first time from tissue culture supernatants of human breast cancer cells (87). PTN is expressed in primary breast cancers and in estrogen receptor-negative breast cancer cell lines (88), and is upregulated in carcinogen induced mammary carcinomas in rats (88).

Nevertheless, *Ptn* expression is also detected in the normal human breast (89) and in the normal mouse mammary gland (90). In the human, *Ptn* is expressed mainly in the alveolar epithelial and myoepithelial cells (89) and it is secreted in human milk and colostrum (91). In the mouse, *Ptn* is expressed in the adipocytes and in the epithelial cells (89) and the differentiation of mammary gland starts after weaning, and hormonal stimuli induces ductal tree proliferation, branching and invasion of the mammary fat pads. Treatment with a monoclonal anti-PTN neutralizing antibody has demonstrated that PTN is required to maintain mammary epithelial cells in a proliferative state and delays ductal outgrowth, branching and terminal end formation. In particular, PTN seems to inhibit differentiation and to delay mammary gland maturation through the inhibition of phospho ERK1/2 signaling (92).

The levels of *Ptn* mRNA also vary during the intense remodeling of mammary gland during pregnancy and lactation. *Ptn* expression is not modified during the first 10 days of pregnancy when ductal epithelial cells proliferate in mammary gland. However, by day 15, when the epithelial ductal cells start undergoing lobular-alveolar differentiation, *Ptn* mRNA levels are downregulated 30-fold. After weaning, during the apoptosis of mammary epithelial cells after lactation, *Ptn* mRNA levels are restored to the ones of non-pregnant animals (92). Moreover, lower *Ptn* mRNA levels were detected in the mammary gland of parous dams when compared to nulliparous dams in rodents (93).

## Pleiotrophin and pancreas: the pancreatic beta cell

Pleiotrophin is highly expressed during embryonic and fetal development in those organs that undergo branching morphogenesis, like the pancreas (75). During the branching ductal morphogenesis of the embryonic pancreas in the mice, PTN modulates cell proliferation and angiogenesis (94). Prior to pancreatic evagination, PTN is located in the areas of vasculogenesis adjacent to the differentiating ductal epithelium. At the embryonic day E11-13 in mice, PTN is localized in the basement membranes of the pancreatic epithelium. With the progress of differentiation, PTN becomes localized only to the undifferentiated “cord” region, and by E18 only to blood vessels. Furthermore, in mouse 3D culture of E11 pancreatic explants, the antisense inhibition of *Ptn* expression impaired the differentiation of endocrine precursors and blunted glucagon and insulin expression (94).

Pancreatic β cells share with neurons that are excitable cells (95) and that retain high levels of *Ptn* expression in the adult. In fact, PTN maintains a high level of expression in the β cells of pancreatic islets of adult rats [10], mice [10] and humans [10]. The expression of *Ptn* in β cells could potentially contribute to adaptive increases in β cell mass as PTN is prominently expressed in Ins^+^ ${\text{Glut}}_{2}^{\text{LO}}$ cells, the immature pancreatic β cells that have multipotential islet endocrine cell lineage potentiality, and retain the proliferative capacity (10).

Although αvβ3 integrin is a PTN receptor that is expressed in the pancreatic islets (96), RPTP β/ζ is the most probable target for PTN in pancreatic islets (10). Treatment with rPTN increased DNA synthesis and upregulated the expression of *Pdx-1* and *insulin* genes in the INS1E rat insulinoma cells suggesting that PTN modulates β cell expansion through RPTP β/ζ and/or other surface binding proteins (10).

On the other hand, the excitability of β cells is dependent of glucose uptake and triggers the opening of the voltage-dependent calcium channels, allowing the entrance of calcium inside the β cell which leads to insulin secretion (97, 98). In previous studies using female *Ptn*^-/-^ mice, glucose tolerance and insulin responsiveness were improved in 3-month-old *Ptn*^-/-^ mice, but in later life these mice become hyperinsulinemic, insulin-resistant and glucose intolerant (11). Therefore, *Ptn* deletion may favor a prediabetic state that increases the susceptibility to develop diabetes in later stages of life. Besides, late pregnant *Ptn* knock out mice are hypoinsulinemic, hyperglycemic and glucose intolerant and have a decreased expression of key proteins involved in glucose and lipid uptake and metabolism (56).

## Conclusions and future perspectives

Pleiotrophin has been widely studied in the field of cancer and much work has been also carried out to analyze its implications in the CNS. In this review, we summarize the main actions of PTN at the main metabolic peripheral organs obtained from the experiments in which pleiotrophin levels are increased (by recombinant pleiotrophin administration and transgenic overexpression) or reduced (by miRNA, siRNA, neutralizing antibodies or *Ptn* deletion).

In the mammary gland, this cytokine favors tumor growth and progression and delays the full development of the tissue. In this line of evidence, blocking PTN with anti-PTN antibody enhanced ductal development in the mammary glands. Pleiotrophin is expressed in the young endocrine pancreas and in the adult pancreas and it is overexpressed during β cell regeneration after streptozotocin treatment. Moreover, the administration of recombinant pleiotrophin in an insulinoma cell line induces β cell expansion and enhances the expression of insulin-related genes. In the muscle, pleiotrophin is overexpressed during tissue regeneration. Transgenic overexpression of *Ptn* in the muscle enhances vascularization and oxidative muscle metabolism through an increment in the activity of citric acid cycle and electron transport system. No evidence is available yet regarding the effects of *Ptn* deletion on muscle oxidative metabolism. However, the actions of PTN on oxidative metabolism and whole-body energy expenditure are also derived from the effects of PTN on adipose tissue and liver. *Ptn* expression increases in preadipocytes during confluence and quickly decreases once differentiation starts. In fact, when adipocyte differentiation starts miR-143 levels start to increase what may repress *Ptn* expression and treatment with rPTN inhibits preadipocyte differentiation. Nevertheless, pleiotrophin deletion impairs fat accumulation and the expansibility of adipose tissue and favors lipolysis of triacylglycerides in the adipose tissue and free fatty acid release. Moreover, pleiotrophin deletion induces browning in white adipose tissue and increases the thermogenic activity of the brown adipose tissue increasing the use of fatty acids instead of carbohydrates for energy production. The role of PTN on hepatic lipid metabolism and development has also been clearly evidenced. PTN expression increases during liver regeneration and development. Additionally, the overexpression of pleiotrophin has also shown to increase fatty acid synthesis and the expression of lipogenic genes. However, *Ptn* deletion decreases both the liver size and the gene expression of proteins involved in lipogenesis, both during late pregnancy and in a HFD-induced obesity mice model, and thus may protect against steatosis.

These findings suggest a role for pleiotrophin in the development of pathologies related to insulin resistance such as aging or diabetes, however, much effort is still needed to fully understand and deepen the knowledge of the field. We believe in the importance of a more profound and further characterization of *Ptn* knock out and transgenic animal models to fully elucidate the implications of this cytokine in pancreas functionality and muscle metabolism.

## Author contributions

All authors contributed to the writing and review of the manuscript. All authors have read and agreed to the published version of the manuscript. The article is an original work, that hasn’t received prior publication and isn’t under consideration for publication elsewhere.
